# [Retracted] MicroRNA-146a protects against intracerebral hemorrhage by inhibiting inflammation and oxidative stress

**DOI:** 10.3892/etm.2025.12970

**Published:** 2025-09-16

**Authors:** Xin Qu, Ning Wang, Weitao Cheng, Yueqiao Xue, Wenjin Chen, Meng Qi

Exp Ther Med 18:3920-3928, 2019; DOI: 10.3892/etm.2019.8060

Following the publication of this paper, it was drawn to the Editor’s attention by a concerned reader that certain of the flow cytometric (FCM) assay data featured in Fig. 4 were strikingly similar to FCM data that had already been submitted for publication to the (same) journal *Experimental and Therapeutic Medicine*, which was written by different authors at different research institutes.

Owing to the fact that the contentious data in the above article had already been submitted for publication elsewhere, the Editor of *Experimental and Therapeutic Medicine* has decided that this paper should be retracted from the Journal. After contacting the authors, they accepted the decision to retract the paper. The Editor apologizes to the readership for any inconvenience caused.

